# Gaps and challenges: WHO treatment recommendations for tobacco cessation and management of substance use disorders in people with severe mental illness

**DOI:** 10.1186/s12888-020-02623-y

**Published:** 2020-05-14

**Authors:** Jayati Das-Munshi, Maya Semrau, Corrado Barbui, Neerja Chowdhary, Petra C. Gronholm, Kavitha Kolappa, Dzmitry Krupchanka, Tarun Dua, Graham Thornicroft

**Affiliations:** 1 Department of Psychological Medicine, Institute of Psychiatry Psychology & Neurosciences, King’s College London, South London & Maudsley NHS-Trust, De Crespigny Park, London, SE5 8AF UK; 2 Centre for Global Health Research, Brighton and Sussex Medical School, Brighton, UK; 3 WHO Collaborating Centre for Research and Training in Mental Health and Service Evaluation, Department of Neuroscience, Biomedicine and Movement Sciences, Section of Psychiatry, University of Verona, Verona, Italy; 4 Department of Mental Health and Substance Abuse, World Health Organization, Geneva, Switzerland; 5 Centre for Global Mental Health, Institute of Psychiatry, Psychology & Neuroscience, King’s College London, London, UK; 6 The Chester M. Pierce, MD Division of Global Psychiatry, Massachusetts General Hospital, Boston, USA

**Keywords:** Severe mental illness, Schizophrenia, Bipolar affective disorders, Depression, Life expectancy, Mortality, Ethnicity, Deprivation, Schizoaffective disorders, Serious mental illness

## Abstract

**Background:**

People with severe mental disorders (SMD) experience premature mortality mostly from preventable physical causes. The World Health Organization (WHO) have recently produced guidelines on the management of physical health conditions in SMD. This paper presents the evidence which led to the recommendations for tobacco cessation and management of substance use disorders in SMD.

**Methods:**

Scoping reviews informed 2 PICO (Population Intervention, Comparator, Outcome) questions relating to tobacco cessation and management of substance use disorders in SMD. Systematic searches led to the identification of systematic reviews with relevant evidence to address these questions. Retrieved evidence was assessed using GRADE methodology, informing the development of guidelines.

**Results:**

One thousand four hundred thirty-four records were identified through systematic searches for SMD and tobacco cessation, of which 4 reviews were included in GRADE tables and 18 reviews in narrative synthesis. For SMD and substance use disorders, 4268 records were identified, of which 4 studies from reviews were included in GRADE tables and 16 studies in narrative synthesis.

People with SMD who use tobacco should be offered combined pharmacological (Varenicline, Bupropion or Nicotine Replacement Therapy) and non-pharmacological interventions such as tailored directive and supportive behavioural interventions. For people with SMD and substance use disorders (drug and/or alcohol), interventions should be considered in accordance with WHO mhGAP guidelines. Prescribers should note potential drug-drug interactions. Recommendation were conditional and based on low/very low certainty of evidence with a scarcity of evidence from low- and middle-income settings.

**Conclusions:**

These guidelines mark an important step towards addressing premature mortality in people with SMD. The dearth of high-quality evidence and evidence from LMIC settings must inform the future research agenda.

Guidelines: https://www.who.int/mental_health/evidence/guidelines_physical_health_and_severe_mental_disorders/en

https://www.who.int/publications-detail/mhgap-intervention-guide%2D%2D-version-2.0

## Background

The severe mental disorders (SMD), defined as schizophrenia-spectrum, psychoses and bipolar disorders as well as moderate to severe depression, are associated with markedly reduced life expectancy [1]. Worldwide, reductions in life expectancy amongst people with SMD are stark, ranging from 11 to 17 years in the UK [2], 15–20 years across Nordic countries [3], and up to 30 years reduced in low- and middle-income country (LMIC) settings such as in Ethiopia [4]. In particular, this decrement in life expectancy has been noted to be increasing over time [5].

Although deaths from suicide and other unnatural causes may be more likely in this group compared to general populations, the majority of deaths are in fact due to preventable physical causes, such as cardiovascular disease, respiratory disorders, cancers and infectious disease [6]. In addition, lowered life expectancy may also be because comorbid substance use disorders (harmful substance use and dependence) are the most prevalent psychiatric conditions associated with SMD. Lifetime alcohol use disorders may affect up to 20% of people with schizophrenia [7] and between 24 to 35% of people with bipolar disorders [8, 9]. Comorbid substance use disorders such as cannabis use disorder [10], opioid and other drug use disorder are also known to be more prevalent in these populations compared with the general population [9]. Tobacco use has also been noted to be elevated more than five-fold in people with schizophrenia compared to reference populations [11, 12] and is a leading preventable cause of death in this group of people. Global successes in reducing tobacco use in the general population have not been mirrored by similar reductions in populations with SMD [11, 13].

A history of substance abuse in populations with SMD has been shown to be associated with an increased risk of death from all-causes and from unnatural causes [14–16]. In addition, findings from a recent study indicated that in general, the presence of substance use disorders (across a broad spectrum of substance types) in SMD was associated with an increased risk of psychiatric admissions, psychiatric emergency department presentations and longer in-patient stays [17]. People with SMDs probably do not just use one substance in particular but are more likely to engage in polysubstance use [17]. Factors which make people with dual diagnoses (comorbid mental and substance use disorders) particularly vulnerable to poor health and social outcomes, include the mutually detrimental effect on the course of illness, its identification, diagnosis and treatment; double stigma and barriers to both mental and physical health care, as well as the contribution of substance use to negative health and social outcomes. For tobacco use, the prevalence of tobacco use in people with SMD is higher, and people with SMD are known to start smoking earlier and smoke more heavily [18] compared with the general population [19]. Potential aetiological pathways for premature mortality in SMD populations with these comorbidities are complex and interlinked. Some basic pathways are summarised in Table 1.

**Table 1:** Risks and consequences associated with mortality for tobacco, alcohol and drug use in SMDs

Tobacco	Alcohol and Drugs
**Systemic factors**	
Lack of data- so that scale of problem in some countries is unclear- allowing the issue to be ignored [47, 48]	Lack of data- so that scale of problem in some countries is unclear- allowing the issue to be ignored^[1, 2]^
Lack of/ or limited funding for healthcare service provision [49]	Lack of/ or limited funding for healthcare service provision^[3]^
Low levels of service provision for people with SMDs to be able to access interventions [48]	Low levels of service provision for people with SMDs to be able to access interventions^[2]^
Lack of training/ capacity building- impacting on ability to deliver interventions, particularly in lower resourced settings [49]	Lack of training/ capacity building- impacting on ability to deliver interventions, particularly in lower resourced settings^[3]^
Vertical approaches to healthcare delivery which lead to ‘silos’ in service provision (mental health and physical health provision not well integrated, or health and social care poorly integrated) [49, 50]	Vertical approaches to healthcare delivery which lead to ‘silos’ in service provision (mental health and physical health provision not well integrated, or health and social care poorly integrated)^[3, 4]^
-	Mental health provision or tackling service provision for dual diagnoses populations is a low priority for government [49]
**-**	Criminal justice (instead of public health) to address people with substance use disorders
**Beliefs/Awareness**	
Healthcare provider belief of futility- that patients will not benefit- leading to lower levels of intervention offered	Healthcare provider belief of futility- that patients will not benefit- leading to lower levels of intervention offered
Healthcare provider- Lack of awareness or knowledge relating to evidence-based interventions and application of these	Healthcare provider- Lack of awareness or knowledge relating to evidence-based interventions and application of these
Healthcare provider belief that smoking cessation may exacerbate mental state or concerns about pharmacotherapy interactions- leading to lower levels of cessation advice and intervention being offered [51].	Beliefs that alcohol and/or drugs are helpful as self-treatment for depression and other mental health conditions
‘Culture’ of smoking in services for people with SMDs which may increase the risk of smoking initiation [52]	-
c-	Lack of awareness (on part of healthcare provider or service user) of treatment need for substance/ alcohol use disorders [48]
**Inequalities**	
In the general population, a social class gradient is observed for tobacco use. May be reflected in people with SMDs who are also more likely to ‘drift’ into lower socioeconomic position	Complex bidirectional associations with unemployment, lower socioeconomic position and other indicators of poverty and exclusion (e.g. homelessness) associated with usage and with poorer physical health and excess mortality
-	Higher risk of social exclusion and ‘extreme inequalities’ for dual diagnosis populations- directly impacting on reduced or delayed access to mental/ physical healthcare [50], also reflected in exclusion from research [39].
-	Impact on mental state- comorbid substance/ alcohol use impacts on severity and remission, increasing the risk of onset, recurrence and reducing chances of recovery. Impact on adherence to treatments.
Respiratory disorders, e.g. COPD leading to pneumonia	Alcohol withdrawal, delirium tremens. Overdose (opioids and other drugs).
Cancers e.g. Lung, other	Acute alcohol/ drug intoxication. Exacerbation of mental state, death through indirect pathways.
Increased susceptibility to infection e.g. TB	Alcoholic hepatitis, pancreatitis, ulcer (gastric, duodenal). Increased risk of range of infections- chest infection, TB, HIV, hepatitis- through multifactorial causes (e.g. injecting drug use)
-	Increased risk-taking behaviours as a result of intoxication with impact on physical health (e.g. infectious diseases, increased risk of STDs)
Modifiable risk factor for dementia in later life [53]	Neurological sequelae and impact on cognition- Wernicke Korsakof’s syndrome, alcohol-related brain damage. Increased risk of accidents- leading to trauma/ head injury (e.g. subdural haemorrhage)
-	Malnutrition
-	Self-harm/ suicidal behaviours secondary to intoxication/ withdrawal

To improve the management of comorbid conditions in adults with SMD and support the reduction of individual health behaviours constituting risk factors for these illnesses, with the aim of decreasing morbidity and premature mortality amongst people with SMD, in 2018 the World Health Organization (WHO) launched guidelines for the “*Management of physical health conditions in adults with severe mental disorders”* [20]. Prior to the launch of these guidelines it was recognised that whereas there are WHO guidelines addressing mental and substance use disorders as well as physical health conditions in general populations, there was an absence of guidelines specifically targeting those with SMD having comorbid conditions. The target audience for the guidelines are health care practitioners across all specialisms and levels of health care system, as well as policy makers, healthcare planners/providers, programme managers, and people living with SMD as well as their families and carers, and organisations representing the interests of people living with SMD.

In this paper, we present the findings of a detailed comprehensive overview of existing systematic reviews on the topic areas of tobacco cessation and management of comorbid substance use disorder in SMD, which eventually led to the recommendations in the WHO guidelines on management of physical health conditions in adults with severe mental health disorders. The full guidelines and supporting materials can be accessed from the WHO website (https://www.who.int/mental_health/evidence/guidelines_physical_health_and_severe_mental_disorders/en/).

## Methods

The methodologies used to inform the WHO recommendations for the management of tobacco and substance use disorders among people with SMD followed the GRADE (Grading of Recommendations Assessment, Development and Evaluation) process [21].

A key outcome of the initial phase in developing the guidelines was in the identification of target areas which eventually informed the a priori research questions which followed the PICO [Population, Intervention, Comparison group, Outcomes] format. The research questions guided which physical health conditions and risk factors were to be addressed in the final disseminated guidelines [20]. This process was informed by scoping reviews and consultation with a Guideline Development Group (GDG) of externally appointed international experts, engaged by the WHO. Selected PICO questions reflected areas of uncertainty which the GDG felt should be prioritised to inform final recommendations. The final research questions for informing systematic evidence searches were then ratified by the WHO Guideline Review Committee (GRC), which led to the formulation of specific research questions relevant to tobacco and substance use disorders among people with SMD (Tables 2 and 3).

**Table 2:** Research questions- tobacco use

*For people with SMD who use tobacco, are pharmacological (including nicotine replacement therapy, bupropion, varenicline) and/or non-pharmacological interventions effective to support tobacco cessation?* **Population/ Intervention / Comparison / Outcome (PICO)** **Population:** People with SMD who use tobacco **Intervention:** • Pharmacological interventions: including nicotine replacement therapy (NRT), bupropion, varenicline • Non-pharmacological interventions **Comparison:** care as usual and/or placebo **Outcomes:** • Critical o Tobacco cessation/abstinence rates o Tobacco consumption rates o Respiratory disease outcomes (COPD, asthma) • Important: o Frequency of adverse events/side-effects

**Table 3:** Research questions- substance (drug and/ or alcohol) use disorders

*For people with SMD and substance (drug and/or alcohol) use disorder, are pharmacological and/or non-pharmacological interventions for substance use disorder effective to support reduction in substance use-related outcomes?* **Population/ Intervention / Comparison / Outcome (PICO)** **Population:** people with SMD and substance (drug and/or alcohol) use disorder **Intervention:** pharmacological and/or non-pharmacological interventions for substance use disorders: - Pharmacological interventions - Non-pharmacological interventions: e.g. motivational interviewing and/or CBT, psychoeducation, brief assessment interview, dual-focus interventions **Comparison:** care as usual / placebo or one treatment vs another **Outcomes:** Critical - Level of consumption - Frequency of use - Abstinence - Relapse rates Important: - Frequency of adverse events / side-effects	

**Fig. 1:**
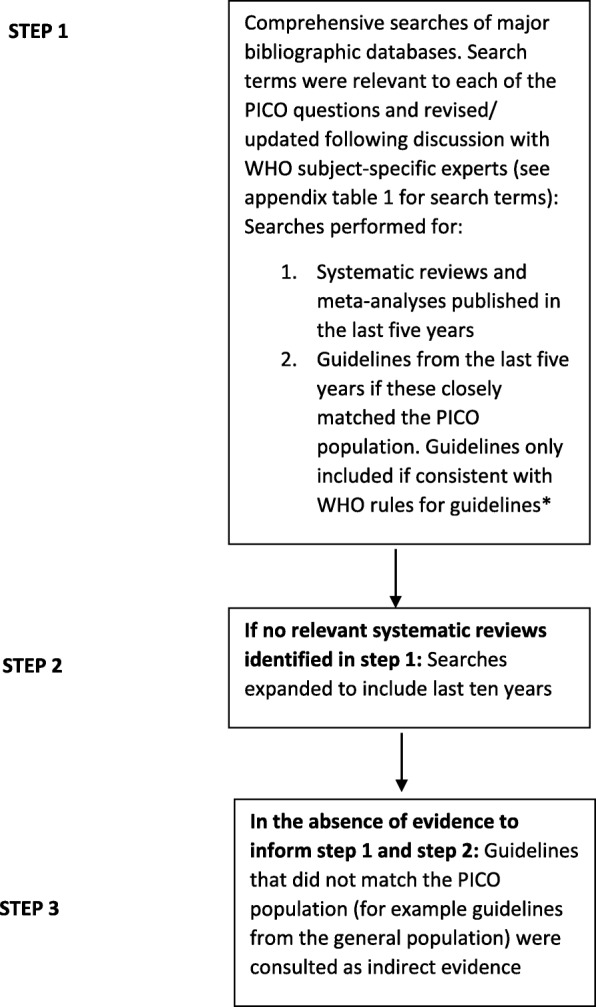
Processes followed to identify direct and indirect evidence for the PICO questions

Figure 1 highlights the comprehensive processes which were followed, leading to the identification of relevant systematic reviews to inform the research questions relating to tobacco cessation, and treatment of substance use disorders in SMD. The retrieval, appraisal and synthesis of evidence closely followed the WHO handbook for guideline development [22]. Databases searched included: the Cochrane Library (including DARE), PubMed/Medline, Embase, Psychinfo, Epistemonikos and the Global Health Library. In addition, where searches had to be expanded (see step 3 in Fig. 1) the National Guideline Clearing House was also searched. Search terms employed for the research questions are displayed in supplementary material, and reflected the majority of substances listed in chapters F10-F19 of the tenth revision of the International Classification of Diseases and Related Health Problems (ICD-10) [23]. (Supplementary material: Table 1); these were informed through consultation with guideline methodologists and subject-specific experts at the WHO. Supplementary searches highlighting relevant drug-drug interactions were also employed (Supplementary material: Table 2). Searches between medicines used for tobacco cessation or treatment of substance use disorders and those used for SMDs were carried out using the drug-drug interaction software Lexi-Interact [24]. Lexi-Interact was selected for its clinical utility and the fact that it scored well on both accuracy and comprehensiveness in a review comparing drug-drug interaction software databases [25]. Searches were performed to February 2018 for the tobacco PICO question and to June 2018 for the substance use disorders PICO question.

Systematic reviews selected for inclusion into GRADE tables conformed to the following inclusion criteria: (1) **Timelines-** Published within the last 5 years, preferably within the last 3 years; (2) **Quality-** Papers included for GRADE assessment had sufficiently high methodological quality ratings on the ‘Assessment of Multiple Systematic Reviews’ tool (AMSTAR) [26–28] (see below for further details); (3) **Relevance-** Retrieved papers were closely relevant to the PICO population. However, where relevant evidence could not be identified these criteria were relaxed, leading to ‘indirect evidence’ to inform recommendations (Fig. 1, step 3). Cochrane reviews or comprehensive meta-analyses and systematic reviews were given preference, wherever possible in this process.

In order to inform the development of evidence based guidelines in a transparent manner, the GRADE approach was used [21]. An advantage of GRADE is that the certainty of the evidence can be summarised and assessment of the evidence can be separate to the strength of the recommendations which inform the final guidelines [21].

Prior to selection for GRADE assessment, retrieved articles had to meet sufficiently high quality ratings on the AMSTAR tool [26–28]. The AMSTAR tool leads to a score across 11 domains according to which the quality of each retrieved systematic review is rated. Papers were initially assessed by a member of the team and then cross-checked by another member of the team (MS, JD, PCG). Systematic reviews fulfilling inclusion criteria with a sufficiently high AMSTAR quality rating (a positive rating on more than 6 out of 11 domains) were then assessed using the GRADE approach using the GRADEpro tool by a member of the team (MS), with all GRADE assessed papers subsequently rated by a second rater (JD and CB). Discordant ratings between team members on the AMSTAR and the GRADE were resolved through discussion in the team. Key attributes of studies relating to each of the PICO questions were extracted from each included study using a structured form by one member of the team and cross-checked by another. WHO guidelines for rating studies in terms of certainty of evidence, according to the GRADE were followed, to assess each study for limitations, inconsistency, indirectness, imprecision and the reporting of bias, leading to a final GRADE assessment of the certainty/confidence of the findings reported in the review [29]. For each included study a relevant summary measure was extracted, which was either a Relative Risk (RR) or Mean Difference (MD).

GRADE evidence profiles for each of the PICOs were presented and discussed over a series of roundtable meetings convened at the WHO in Geneva in May 2018. GDG members were selected internationally across UN member states for their expertise within the topic areas. In addition, the meetings were also attended by a guideline methodologist, the evidence review team and the WHO secretariat. The final recommendations resulted from a consideration of the background evidence for each of the PICO questions, summarised as GRADE profiles and the certainty of evidence for these, as well as taking into consideration other aspects such as whether the problem was considered a priority, how substantial desirable and undesirable anticipated effects were, whether the balance between desirable/undesirable effects favoured the intervention over the comparator, the value attached to the outcomes and the certainty of evidence relating to likely resource requirements, cost effectiveness, impact on health equity, acceptability and feasibility of the intervention. In addition the acceptability of the intervention to healthcare providers in LMICs, feasibility of the intervention and the impact of the intervention on equity and human rights were considered.

## Results

After consultation with the GDG and WHO GRC agreed research questions specific to tobacco cessation and substance use disorders were:

1. For people with SMD who use tobacco, are pharmacological (including nicotine replacement therapy, bupropion, varenicline) and/or non-pharmacological interventions effective to support tobacco cessation?
2. For people with SMD and substance (drug and/or alcohol) use disorder, are pharmacological and/or non-pharmacological interventions for substance use disorder effective to support reduction in substance use-related outcomes?

**Fig. 2:**
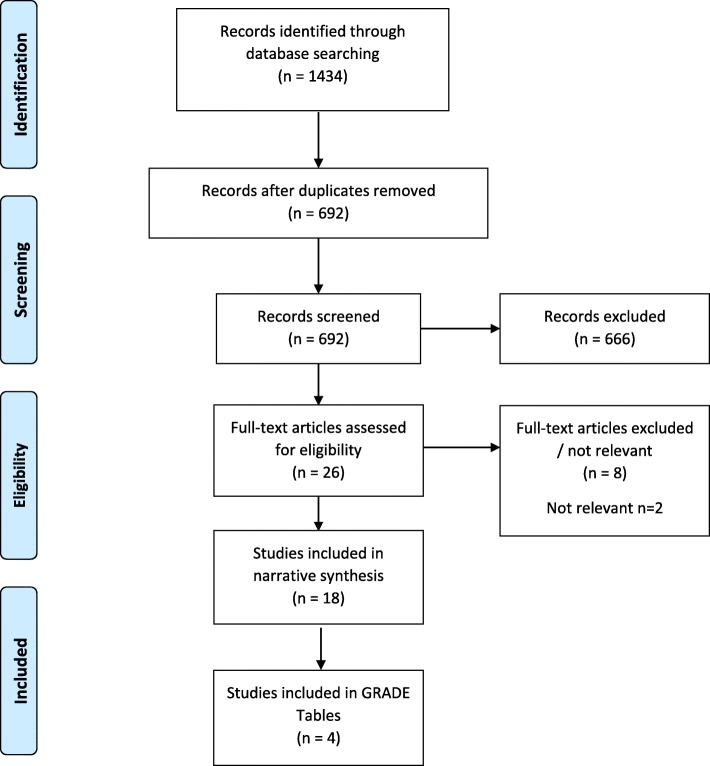
PRISMA Flow Diagram for systematic review of the reviews: SMD and tobacco cessation

**Fig. 3:**
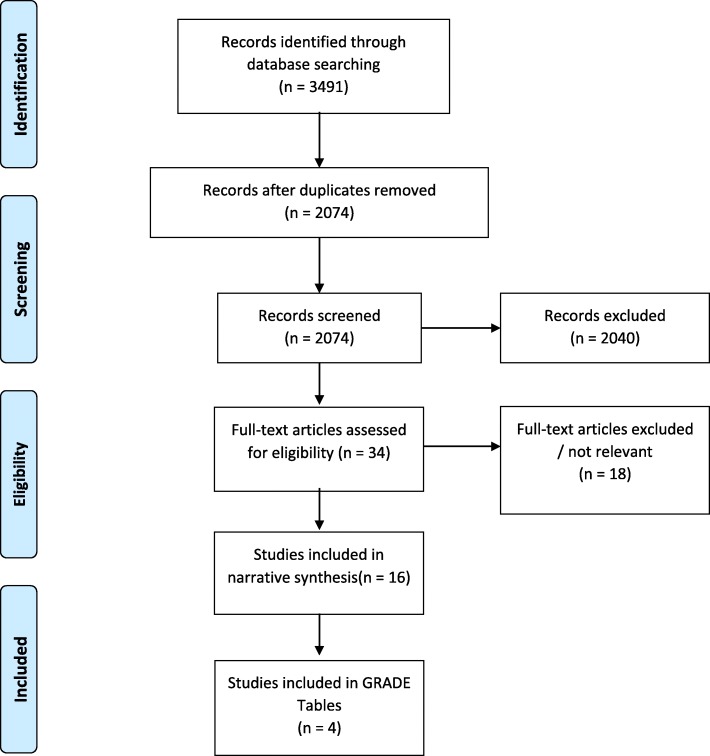
PRISMA Flow Diagram for systematic review of the reviews: SMD and substance use disorders

In total 1434 records were initially identified through the systematic searches for SMD and tobacco cessation; after screening for eligibility and removal of duplicates, 4 reviews were included in the GRADE tables for this PICO with 18 reviews in total contributing evidence through narrative synthesis. For SMD and substance use disorders, a total of 4268 records were identified. After screening and checking against eligibility criteria, 4 studies were included in the GRADE tables on this topic with a total of 16 studies included in the narrative synthesis. Figures 2 and 3 display PRISMA flow charts of relevant articles retrieved for SMD and tobacco use and with substance use disorders, respectively.

For tobacco use in SMD, GRADE evidence profiles were compiled for: the use of Buproprion, Varenicline and Nicotine Replacement Therapies (NRT) (all versus placebo). In addition, GRADE profiles for non-pharmacological interventions (which included: motivational enhancement, psychoeducational approaches, Cognitive Behavioural Therapy (CBT)), supplementing NRT were compared to standard care approaches, and the use of contingent reinforcement (using money/money plus NRT) compared to care-as-usual was assessed with GRADE [30–33] (For full recommendations with supporting evidence, including relevant drug-drug interactions for Buproprion, Varenicline and NRT see: https://www.who.int/mental_health/evidence/guidelines_physical_health_and_severe_mental_disorders/en/). The GDG recommended combination pharmacological with behavioural interventions, as behavioural interventions alone have been shown to result in a relatively low abstinence rate for tobacco use in SMD.

The certainty of evidence derived from GRADE, relating to specialised smoking cessation interventions versus standard approaches in people with SMD, was very low. There was insufficient evidence to suggest the superiority of specialised smoking interventions over standard smoking cessation approaches for SMD populations. In addition, the certainty of evidence relating to contingency reinforcement approaches compared with care-as-usual for tobacco cessation in SMD populations was very low.

Pharmacological interventions identified for tobacco cessation in SMD populations were: NRT, Bupropion and Varenicline. Evidence for the efficacy of these interventions in SMD populations mostly derived from high income settings with a few exceptions (e.g. studies for Bupropion which had been conducted in China and Iran as well as in the USA). These pharmacological interventions for tobacco cessation are already recommended by the WHO in general populations, although only NRT is on the WHO essential medicines list [23]. Searches of pharmacological interactions indicated the possibility of interactions between Bupropion and psychotropic medications commonly prescribed in SMD, particularly related to lowering seizure threshold and enzyme inhibition or induction (see https://www.who.int/mental_health/evidence/guidelines_physical_health_and_severe_mental_disorders/en/for full list of interactions).

For substance use disorders and severe mental disorders, assessment of evidence using the GRADE approach included a review of evidence relating to psychological interventions such as CBT plus motivation interviewing (MI) versus care-as-usual, CBT versus care-as-usual, MI versus care-as-usual and contingency management versus care-as-usual for people with SMD and substance use disorders [34]. Brief interventions, specifically delivered in four or fewer sessions [35], were also assessed. Although these types of interventions may have a basis simply in providing education and advice [35], the brief interventions which were identified and assessed according to GRADE for these guidelines all compared motivational interviewing with CBT approaches, delivered over shorter time frames [35]. In addition, evidence relating to the efficacy of antipsychotic medications in reducing psychotic symptoms alongside other outcomes such as frequency of substance use, in dual diagnoses populations were also assessed [36, 37] as well the prescribing of antidepressants in depression comorbid with alcohol use disorders to improve outcomes [38].

All of the main recommendations relating to each of the PICO questions are presented in Table 4. For dual diagnoses populations, there was a lack of evidence to support the superiority of any of the psychological interventions in improving outcomes related to SMD comorbid with substance use disorders. Furthermore, the review team were unable to identify any studies which had specifically assessed these populations within LMIC settings, further limiting generalisabilty. Of those studies retrieved, most were of very low certainty. The GDG reflected that the relative lack of evidence to support the efficacy of these interventions in people with SMD comorbid with substance use disorders may partly be due to these populations being more likely to be excluded from research [39].

**Table 4:** WHO Recommendations- the management of tobacco use, substance use disorders in people with severe mental disorders

Question	Recommendation	Strength of recommendation
**For people with SMD who use tobacco, are pharmacological (including nicotine replacement therapy, bupropion, varenicline) and/or nonpharmacological interventions effective to support tobacco cessation?**	In people with severe mental disorders, combined pharmacological and non-pharmacological interventions may be considered in accordance with the WHO training package (Strengthening health systems for treating tobacco dependence in primary care. Building capacity for tobacco control: training package) (http://www. who.int/tobacco/publications/building_capacity/training_package/treatingtobaccodependence/en/).	Conditional; quality of evidence- very low
	In people with severe mental disorders, a directive and supportive behavioural intervention programme may be considered and should be tailored to the needs of the population.	Conditional; quality of evidence- very low
	In people with severe mental disorders, varenicline, bupropion and nicotine replacement therapy may be considered for tobacco cessation.	Conditional; quality of evidence- very low
**Best practice recommendation**	Prescribers should take into account potential interactions between buproprion and varenicline with psychotropic medications as well as possible contra-indications.	
For people with SMD and substance (drug and/or alcohol) use disorder, are pharmacological and/or non-pharmacological interventions for substance use disorder effective to support reduction in substance use-related outcomes?	For people with severe mental disorders and comorbid substance use disorders (drug and/or alcohol) interventions should be considered in accordance with the WHO mhGAP guidelines.	Conditional; quality of evidence- low
	Non-pharmacological interventions (e.g. motivational interviewing) may be considered and tailored to the needs of people with severe mental disorders and substance use disorders.	Conditional; quality of evidence- very low
**Best practice recommendation**	Prescribers should take into account the potential for drug-drug interactions between medicines used for treatment of substance use disorders and severe mental disorders.	
	**Additional considerations:** • There was some non-consistent evidence to indicate effectiveness of motivational interviewing in reducing cannabis and alcohol use in dual diagnoses populations in terms of level of consumption, frequency of use, and abstinence. • Findings from one study identified from reviews indicated that contingency management for substance use may be beneficial in terms of frequency of use (stimulants and alcohol) and non-abstinence (stimulants) • In populations with depression and comorbid alcohol use disorders there is some indication that antidepressants may be more effective than placebo in reducing number of drinks on drinking days or increasing the number of people abstinent. • The GDG also highlighted that, for injecting drug users, testing for Hepatitis B and C and vaccination for Hepatitis A and B should be considered.	

In general, the assessment of evidence using GRADE methods indicated low to very low certainty evidence from randomised controlled trials of pharmacological interventions for the management of mental disorders (whether through the use of antipsychotics or antidepressants), which did not indicate the superiority of any of the surveyed medications, when prescribed for people with SMD comorbid with substance use disorders [36–38]. Moderate side effects were noted for these interventions, which need to be taken into account when prescribing for this patient population. In addition, it was noted that medicines which may be used for the management of opioid use disorders such as Methadone and Buprenorphine have interactions with many of the commonly used psychotropic medications, including cardiac effects such as QTc prolongation, central nervous system depression and serotonergic effects (see Annex 6 of guidelines for details: https://apps.who.int/iris/bitstream/handle/10665/275718/9789241550383-eng.pdf?ua=1).

For both comorbid tobacco use and substance use disorders, where retrieved evidence was of very low certainty, the expertise of the international GDG was sought, who applied their expertise to the topic area. As a result of the low/very low certainty of evidence retrieved, resultant recommendations were conditional. A ‘conditional’ recommendation by the GDG indicates that GDG members concluded that beneficial effects of the intervention probably outweighed undesirable effects but with insufficient evidence for the GDG to support a ‘strong’ recommendation (with ‘strong’ recommendations indicating that the GDG felt confident that beneficial effects outweighed undesirable effects for the recommended intervention). For people with SMD and substance (drug and/or alcohol) use disorder, the low certainty of evidence led to the recommendation that the mhGAP guidelines for the management of substance use disorders should be followed (Table 4).

The full GRADE evidence profiles are displayed in the supplementary materials (supplementary tables 1–2) and can also be accessed online. PRISMA checklist has also been provided in supplementary materials (see additional material: PRISMA checklist).

## Discussion

These evidence-based recommendations, based on detailed and comprehensive reviews of systematic reviews, as well as consultation with an international body of experts and WHO specialists, represent a positive and important step towards tackling the 15–20 year reduction in life expectancy, experienced by people with SMD compared to the general population, globally. These guidelines highlight the need to adequately manage tobacco and other substance use disorders in people with SMDs, alongside optimally managing the mental disorder.

Evidence synthesis highlighted a general lack of high-quality evidence detailing effective interventions for tobacco cessation in SMD and/or for dual diagnoses populations. This reflects a systematic exclusion of people with SMD and/or dual diagnoses from clinical trials, despite evidence indicating that mental disorders are highly comorbid with substance use. There is a need to consider and include these populations in future research [39].

Do the guidelines go far enough? The guidelines retain a practical emphasis to inform clinicians, healthcare providers and other professional groups on best-practice recommendations and acknowledge the importance of wider multi-level interventional frameworks to address the inequalities impacting on SMD populations [40]. Within this framework, a consideration of health system factors as well as broader social determinants which include social support, stigma and attempts to reduce social exclusion play a major role [40]. In addition, although not directly addressed by the guidelines, public health actions to prevention implemented at country-level form the backdrop to recommended interventions at a whole population-level [41], irrespective of group-specific evidence; for example recommended interventions for tobacco cessation or harmful alcohol use could be read within the context of country-level increased taxation/pricing policies on tobacco or alcohol, restrictions on the availability of alcohol, measures to restrict drink-driving, restricted tobacco or alcohol advertising as well as population-level educational campaigns on tobacco cessation, and access to screening and brief interventions [42, 43] or other cost effective interventions [44]. In addition, the guidelines should be read in conjunction with public health/systemic interventions at country-level to address and support population-level mental health [45].

Our searches revealed a scarcity of evidence particularly relating to dual diagnoses populations, which impacted on the ability to make strong recommendations relevant to people with SMD and comorbid substance use. The scarcity of good quality evidence to inform the recommendations reflects the experience of authors of a previous systematic review, whereby it was found that more than half of clinical randomised controlled trials on the pharmacological treatment of opioid dependence excluded people with psychiatric disorders [39]. The systematic exclusion of people with mental disorders from randomised controlled trials has also been noted in one other review in which the authors assessed the presence of psychiatric exclusion criteria in randomised controlled trials [46]. The exclusion of people with mental disorders from trials may in part be due to a number of factors, including trialists’ concerns that decisional capacity to take part is more likely to be impaired in people with SMDs, or concerns that the stress or unintended consequence of taking part in a trial may lead to an exacerbation of mental disorder [46]. In addition, pharmaceutical companies may stipulate extensive exclusion criteria to ensure a smoother pathway to regulation and approval for pharmaceutical products [46]. However, these practices lead to “scientific neglect” [46], and as we have highlighted in this paper, serve to perpetuate the inequalities which people with SMDs experience further. For those systematic reviews which were retrieved, there was also an absence of high-quality evidence relating to psychological interventions to address substance use disorders in dual diagnosis populations. This presents a major limitation, as there is a high co-morbidity of psychiatric and substance use disorders in clinical practice, and for practical purposes it is difficult to address one without the other. In future, research which actively includes people with SMD and comorbid substance use are needed particularly to avoid perpetuating further social exclusion and marginalisation.

Most of the evidence which informed the development of the guidelines came from well-resourced settings. This may mean that specific issues relevant to low resource settings may impact on implementation. Issues relating to cost and capacity will need to be taken into account for some recommended interventions. The availability of certain medications- such as Varenicline (which does not currently appear in the WHO essential medicines list) may be restricted in certain contexts, although other interventions (such as NRT) are more widely available. Other factors relating to acceptability of the guidelines and longer term sustainability across countries will need to be monitored. Future guidelines may reflect feedback from people on the ground at the forefront of implementing these guidelines on tobacco use and substance use disorders in SMDs- for example following feedback from health care practitioners, policy makers and public health practitioners.

## Conclusions

Tobacco use and substance use disorders play an important role in heightening the risk of premature mortality in people with SMDs. Our search of the evidence highlighted gaps in the evidence base, which may in part be due to the systematic exclusion of people with SMDs from clinical trials. Despite the challenges described in this paper, these guidelines may mark an important step towards addressing premature mortality in people with SMD. The recommendations may help to inform policy and decision makers globally and in LMIC settings in ensuring more equitable access to tobacco cessation and substance use disorder services for these populations. However the dearth of high-quality evidence and evidence from LMIC settings must inform the future research agenda.

## Supplementary information

**Additional file 1: Table S1.** Search terms. Search terms for each PICO question.

**Additional file 2: Table S2.** Additional search strategies used to identify relevant drug-drug interactions. Search terms specific to potential drug-drug interactions identified in each PICO question.

**Additional file 3: Table S3.** Overview of systematic review searches. Overview of search strategies detailing number of reviews identified.

**Additional file 4.** PRISMA 2009 Checklist

## Data Availability

All supporting documents which informed the development of this manuscript and the guidelines are freely available through the web links provided in this manuscript or through contacting the authors.

## Abbreviations

PICO: Population Intervention, Comparator, Outcome
WHO: World Health Organization
LMIC: Low and middle income country
GRADE: Grading of Recommendations Assessment, Development and Evaluation
NRT: Nicotine Replacement Therapies
MI: Motivation Interviewing
CBT: Cognitive Behavioural Therapy
SMD: severe mental disorders
AMSTAR: A MeaSurement Tool to Assess systematic Reviews
DARE: The Database of Abstracts of Reviews of Effects
WHO GRC: World Health Organization Guidelines Review Committee
