# Correction to: Gaps and challenges: WHO treatment recommendations for tobacco cessation and management of substance use disorders in people with severe mental illness

**DOI:** 10.1186/s12888-020-02684-z

**Published:** 2020-06-23

**Authors:** Jayati Das-Munshi, Maya Semrau, Corrado Barbui, Neerja Chowdhary, Petra C. Gronholm, Kavitha Kolappa, Dzmitry Krupchanka, Tarun Dua, Graham Thornicroft

**Affiliations:** 1 Department of Psychological Medicine, Institute of Psychiatry Psychology & Neurosciences, King’s College London, South London & Maudsley NHS-Trust, De Crespigny Park, London, SE5 8AF UK; 2 Centre for Global Health Research, Brighton and Sussex Medical School, Brighton, UK; 3 WHO Collaborating Centre for Research and Training in Mental Health and Service Evaluation, Department of Neuroscience, Biomedicine and Movement Sciences, Section of Psychiatry, University of Verona, Verona, Italy; 4 Department of Mental Health and Substance Abuse, World Health Organization, Geneva, Switzerland; 5 Centre for Global Mental Health, Institute of Psychiatry, Psychology & Neuroscience, King’s College London, London, UK; 6 The Chester M. Pierce, MD Division of Global Psychiatry, Massachusetts General Hospital, Boston, USA

**Correction to: BMC Psychiatry (2020) 20:237**

**https://doi.org/10.1186/s12888-020-02623-y**

Following publication of the original article [1], the authors identified an error in Table 1. The headings ‘social exclusion’, ‘mental state impact’ and ‘physical health impact’ were missing.

The original article has been corrected.
