# Supplementary material for: Gaps and challenges: WHO treatment recommendations for tobacco cessation and management of substance use disorders in people with severe mental illness
Source: BMC Psychiatry. 2020 May 14;20:237. doi: 10.1186/s12888-020-02623-y (PMC7227317; doi:10.1186/s12888-020-02623-y)
Supplement: Supplementary file 1 — Additional file 1: Table S1. Search terms. Search terms for each PICO question. [file 12888_2020_2623_MOESM1_ESM.docx]

**TABLE 2: ADDITIONAL SEARCH STRATEGIES TO IDENTIFY RELEVANT DRUG-DRUG INTERACTIONS**

| **Drug-drug interactions search strategy (both PICOs)**  Drug-drug interaction searches were conducted between medicines relevant for each PICO and medicines used for SMD. The following process was employed for the searches:   1. Medicines of interest were identified for each PICO by referring to relevant sections of the 2017 WHO Model List of Essential Medicines (EML), as well as prior WHO Guidelines and WHO Mental Health Gap Action Programme Intervention Guide (mhGAP-IG) where applicable. Physical health medicines were limited in scope to those used on a routine basis, rather than emergently. Technical consultation was also sought with relevant departments of WHO. Pharmacological interventions recommended in the forthcoming guidelines were also included. 2. Medicines used for SMD were limited to those included in the WHO mhGAP-IG and/or the 2017 WHO EML. 3. Searches between both lists (medicines relevant for each PICO and medicines used for SMD) were run using the drug-drug interaction software Lexi-Interact. Lexi-Interact was chosen as it is commonly used in clinical practice and scored well on accuracy and comprehensiveness in a recent review comparing 5 drug-drug interaction engines^^[[1]](#footnote-1)^^. 4. Search results for each PICO were summarized and formulated into a narrative synthesis, as well as a table coded by interaction severity and with annotations for review of the GDG. |
| --- |

1. Kheshti R, Aalipour M, Namazi S. “A comparison of five common drug-drug interaction software programs regarding accuracy and comprehensiveness.” Journal of Research in Pharmacy Practice. 2016; 5: 257-263. [↑](#footnote-ref-1)
