# Supplementary material for: Gaps and challenges: WHO treatment recommendations for tobacco cessation and management of substance use disorders in people with severe mental illness
Source: BMC Psychiatry. 2020 May 14;20:237. doi: 10.1186/s12888-020-02623-y (PMC7227317; doi:10.1186/s12888-020-02623-y)
Supplement: Supplementary file 2 — Additional file 2: Table S2. Additional search strategies used to identify relevant drug-drug interactions. Search terms specific to potential drug-drug interactions identified in each PICO question. [file 12888_2020_2623_MOESM2_ESM.docx]

**SUPPLEMENTARY MATERIAL**

**TABLE 1: SEARCH TERMS**

| **For people with SMD who use tobacco, are pharmacological (including nicotine replacement therapy, bupropion, varenicline) and/or non-pharmacological interventions effective to support tobacco cessation?**  **Pharmacological interventions:**  Search #1: Severe mental disorder  (exp Mental Disorders/ OR exp Psychotic Disorders/ OR exp Bipolar and Related Disorders/ OR exp Depressive Disorder) OR  ((Mental AND (disorder* OR disabilit* OR illness OR “health condition” OR “health problem” OR distress)) OR "psychological distress" OR "psychiatric disorder") OR  ((schizophrenia OR schizophrenic) OR Schizotyp* OR ((Delusional OR paranoid) AND disorder*) OR hallucination* OR Psychotic OR Schizoaffective OR psychosis) OR  (((manic OR bipolar OR mood) AND disorder*) OR (depressive AND (disorder* OR episode*)) OR "depressive symptom*" OR hypomania OR mania* OR ((major OR psychotic OR disorder*) AND depression))  Search #2: smoking  (exp Smoking/ OR exp Smokers/ OR exp Tobacco Smoking/ OR exp Tobacco Use/ OR exp Tobacco/ OR exp Nicotine/) OR  (smok* OR tobacco OR nicotin* OR cigarette*)  Search #3: pharmacological tobacco cessation interventions  (exp Smoking Cessation/ OR exp Smoking Reduction/ OR exp Tobacco Use Cessation/ OR exp Tobacco Use Cessation Products/ OR exp Bupropion/ OR exp Varenicline/ OR exp Nicotine Chewing Gum) OR  (exp Drug Therapy/ OR exp Pharmacology/ OR exp Psychopharmacology/ OR exp Metabolic Side Effects of Drugs and Substances/ OR exp Pharmacologic Actions/ OR exp Drug Effects/ OR exp Psychotropic Drugs/) OR  ((drug* OR pharmac* OR medic* OR psychotrop*) AND (intervent* OR therap* OR treat* OR care OR servic*)) OR (drug* OR medic*) OR  ((smok* OR tobacco OR nicotin* OR cigarette*) AND (cessation OR reduc* OR abst*)) OR  (bupropion OR varenicline OR “nicotine replacement therapy” OR “nicotine gum” OR “nicotine patch”)  **Non-pharmacological interventions:**  Search #1: Severe mental disorder  (exp Mental Disorders/ OR exp Psychotic Disorders/ OR exp Bipolar and Related Disorders/ OR exp Depressive Disorder) OR  ((Mental AND (disorder* OR disabilit* OR illness OR “health condition” OR “health problem” OR distress)) OR "psychological distress" OR "psychiatric disorder") OR  ((schizophrenia OR schizophrenic) OR Schizotyp* OR ((Delusional OR paranoid) AND disorder*) OR hallucination* OR Psychotic OR Schizoaffective OR psychosis) OR  (((manic OR bipolar OR mood) AND disorder*) OR (depressive AND (disorder* OR episode*)) OR "depressive symptom*" OR hypomania OR mania* OR ((major OR psychotic OR disorder*) AND depression))  Search #2: smoking  (exp Smoking/ OR exp Smokers/ OR exp Tobacco Smoking/ OR exp Tobacco Use/ OR exp Tobacco/ OR exp Nicotine) OR  (smok* OR tobacco OR nicotin* OR cigarette*)  Search #3: non-pharmacological tobacco cessation interventions  (exp Smoking Cessation/ OR exp Smoking Reduction/ OR exp Tobacco Use Cessation/OR exp Tobacco Use Cessation Products/) OR  (exp Exercise Therapy/ OR exp Therapy/ OR exp Therapeutics/ OR exp Family Therapy/ OR exp Psychotherapy/ OR exp Cognitive Therapy/ OR exp Behaviour Therapy/ OR exp Counseling/ OR exp Mental Health Services/ OR exp Problem Based Learning/ OR exp Problem Solving/) OR  ((psychosocial OR psycho* OR lifestyle* OR cognit* OR behaviour* OR behavior* OR non-pharmac*) AND (intervent* OR therap* OR treat* OR care OR servic*)) OR  (“problem solving” OR psychoeducation OR couns*) OR  ((smok* OR tobacco OR nicotin* OR cigarette*) AND (cessation OR reduc* OR abst*))  **For people with SMD and substance (drug and/or alcohol) use disorder, are pharmacological and/or non-pharmacological interventions for substance use disorder effective to support reduction in substance use-related outcomes?**  **Pharmacological interventions:**  Search #1: Severe mental disorder  (exp Mental Disorders/ OR exp Psychotic Disorders/ OR exp Bipolar and Related Disorders/ OR exp Depressive Disorder) OR  ((Mental AND (disorder* OR disabilit* OR illness OR “health condition” OR “health problem” OR distress)) OR "psychological distress" OR "psychiatric disorder") OR  ((schizophrenia OR schizophrenic) OR Schizotyp* OR ((Delusional OR paranoid) AND disorder*) OR hallucination* OR Psychotic OR Schizoaffective OR psychosis) OR  (((manic OR bipolar OR mood) AND disorder*) OR (depressive AND (disorder* OR episode*)) OR "depressive symptom*" OR hypomania OR mania* OR ((major OR psychotic OR disorder*) AND depression))  Search #2: Substance use disorder  (exp Narcotics/ OR exp Substance-Related Disorders/ OR exp Alcoholism/ OR exp Alcoholics/ OR exp Alcohol Related-Disorders/ OR exp Drug Users/ OR exp Drug Misuse/ OR exp Street Drugs/ OR exp Nonprescription Drugs/ OR exp Drug-Seeking Behaviour/) OR  ("drug abuse" OR "drug addict*" OR "drug depend*” OR "drug withdrawal" OR "drug misuse") OR  ("addictive disease*" OR "addictive disorder*" OR addiction OR addictive OR "substance abuse" OR “substance misuse” OR "withdrawal syndrome" OR psychoactive* harmful use) OR  ("alcoholic patient*" OR "alcoholic subject*" OR alcoholism OR "alcohol depend*" OR "fetal alcohol*" OR "prenatal alcohol*" OR "chronic ethanol*" OR "chronic* alcohol*" OR "alcohol withdrawal" OR "ethanol withdrawal" OR “excessive alcohol consumption” OR “alcohol use disorder” OR “alcohol misuse” OR “alcohol abuse”) OR  ((cocaine OR heroin OR cannabis OR marijuana OR mdma OR Methylenedioxymethamphetamin* OR ecstasy OR morphine* OR amphetamin* OR methamphetamin* OR opioid* OR opiat* OR “prescription drug*” OR “illegal drug*” OR “illicit drug*" OR “street drug” OR benzodiazepin* OR tranquiliz* OR narcot* OR methadone OR fentanyl OR mushrooms OR glue OR inhalant)) AND (abuse OR misuse OR depend* OR addict* OR withdrawal OR overdose OR intoxication OR harmful use OR)) OR methadone DUAL diagnos*  Injecting drug use (IDU$1 OR IVDU$1 OR PWID$1 OR “injecting drug” OR “intravenous drug” OR “injecting substance” OR “intravenous substance” OR exp substance abuse, intravenous/)  Search #3: pharmacological interventions for substance use disorders  (exp Substance Abuse Treatment Centers/ OR exp Alcohol Abstinence/) OR  (exp Drug Therapy/ OR exp Pharmacology/ OR exp Psychopharmacology/ OR exp Metabolic Side Effects of Drugs and Substances/ OR exp Pharmacologic Actions/ OR exp Drug Effects/ OR exp Psychotropic Drugs/) OR  ((drug* OR pharmac* OR medic* OR psychotrop*) AND (intervent* OR therap* OR treat* OR care OR servic)) OR (drug* OR medic*)  Methadone, buprenorphine, naloxone, naltrexone, disulfiram, nalmefene, thiamine, clonidine, lofexidine, acamprosate, baclofen  Opioid agonist maintenance treatment (OST OR MMT OR BMT OR “opioid substitution treatment” OR “methadone” OR “methadone maintenance” OR “buprenorphine” OR “buprenorphine maintenance” OR “opioid replacement” OR exp buprenorphine/ OR exp methadone/ OR exp opiate substitution treatment/ OR exp buprenorphine, naloxone drug combination/  **Non-pharmacological interventions:**  Search #1: Severe mental disorder  (exp Mental Disorders/ OR exp Psychotic Disorders/ OR exp Bipolar and Related Disorders/ OR exp Depressive Disorder) OR  ((Mental AND (disorder* OR disabilit* OR illness OR “health condition” OR “health problem” OR distress)) OR "psychological distress" OR "psychiatric disorder") OR  ((schizophrenia OR schizophrenic) OR Schizotyp* OR ((Delusional OR paranoid) AND disorder*) OR hallucination* OR Psychotic OR Schizoaffective OR psychosis) OR  (((manic OR bipolar OR mood) AND disorder*) OR (depressive AND (disorder* OR episode*)) OR "depressive symptom*" OR hypomania OR mania* OR ((major OR psychotic OR disorder*) AND depression))  Search #2: Substance use disorder  (exp Narcotics/ OR exp Substance-Related Disorders/ OR exp Alcoholism/ OR exp Alcoholics/ OR exp Alcohol Related-Disorders/ OR exp Drug Users/ OR exp Drug Misuse/ OR exp Street Drugs/ OR exp Nonprescription Drugs/ OR exp Drug-Seeking Behaviour/) OR  ("drug abuse" OR "drug addict*" OR "drug depend*” OR "drug withdrawal" OR "drug misuse") OR  ("addictive disease*" OR "addictive disorder*" OR addiction OR addictive OR "substance abuse" OR “substance misuse” OR "withdrawal syndrome" OR psychoactive*) OR  ("alcoholic patient*" OR "alcoholic subject*" OR alcoholism OR "alcohol depend*" OR "fetal alcohol*" OR "prenatal alcohol*" OR "chronic ethanol*" OR "chronic* alcohol*" OR "alcohol withdrawal" OR "ethanol withdrawal" OR “excessive alcohol consumption” OR “alcohol use disorder” OR “alcohol misuse” OR “alcohol abuse”) OR  ((cocaine OR heroin OR cannabis OR marijuana OR mdma OR ecstasy OR morphine* OR amphetamin* OR opioid* OR opiat* OR “prescription drug*” OR “illegal drug*” OR “illicit drug*" OR “street drug” OR benzodiazepin* OR tranquiliz* OR narcot*) AND (abuse OR misuse OR depend* OR addict* OR withdrawal)) OR methadone  Search #3: non-pharmacological interventions for substance use disorders  (exp Substance Abuse Treatment Centers/ OR exp Alcohol Abstinence/ OR exp Needle-Exchange Programs/) OR  (exp Exercise Therapy/ OR exp Occupational Therapy/ OR exp Therapy/ OR exp Therapeutics/ OR exp Family Therapy/ OR exp Psychotherapy/ OR exp Cognitive Therapy/ OR exp Behaviour Therapy/ OR exp Counseling/ OR exp Mental Health Services/ OR exp Problem Based Learning/ OR exp Problem Solving/) OR  ((psychosocial OR psycho* OR lifestyle* OR cognit* OR behaviour* OR behavior* OR non-pharmac*) AND (intervent* OR therap* OR treat* OR care OR servic*)) OR  (“problem solving” OR psychoeducation OR couns*) OR  (“motivational interviewing” (motivational enhancement therapy (MET)) OR CBT OR “cognitive behavioural therapy” OR “cognitive behavioral therapy” OR “brief assessment interview” OR “contingency management” OR “social skills training” OR “relapse prevention” OR “case management” OR “assertive community treatment” OR “family interventions”)  SBIRT – (screening and brief interventions  Psychoeducation  Outreach, Residential programmes, recovery management,  Mutual self-help group (Alcoholic Anonimous, Narcotic Anonimous)  Harm reduction (NSP$1 or NSEP$1 OR “needle syringe program$” OR “needle syringe exchange program$” OR “needle exchange$1” OR “syringe exchange $1” OR exp needle-exchange programs/ OR exp harm reduction/)  **Filters used across all searches:**   - 1. Systematic reviews / meta-analyses / guidelines where relevant (if these closely matched the population to whom the PICO applied to and adhered to the WHO rules for guidelines)   2. Humans   3. Publication within last 5 years, unless searches has to be expanded (see Figure 1- step 2)   4. No language restrictions |
| --- |
