# Supplementary material for: Gaps and challenges: WHO treatment recommendations for tobacco cessation and management of substance use disorders in people with severe mental illness
Source: BMC Psychiatry. 2020 May 14;20:237. doi: 10.1186/s12888-020-02623-y (PMC7227317; doi:10.1186/s12888-020-02623-y)
Supplement: Supplementary file 3 — Additional file 3: Table S3. Overview of systematic review searches. Overview of search strategies detailing number of reviews identified. [file 12888_2020_2623_MOESM3_ESM.docx]

**Table 3****: Overview of systematic review searches**

| The restrictions/filters used in the systematic searches were as follows:   - Only systematic reviews (including meta-analyses) or guidelines were included at this stage. Guidelines have only been included if they closely matched the population to whom the PICO applied and if they adhered to the WHO rules for guidelines. - Only systematic reviews/guidelines that had published in the last five years were included. - Humans only were included. - The ‘Advanced search’ option was employed where possible. |
| --- |

**Tobacco cessation**

*For people with SMD who are smokers, are pharmacological (including nicotine replacement therapy, bupropion, varenicline) interventions effective to support tobacco cessation?*

| **Database searched** | **Date searched** | **No. of papers** | **Combined number, excluding duplicates** | **No. of relevant Cochrane reviews** | **No. of relevant systematic reviews** | **No. of relevant guidelines** |
| --- | --- | --- | --- | --- | --- | --- |
| Cochrane (& DARE) | 16.02.18 | 168 | 727 | 2  (+ 1 protocol) | 16 | 5 |
| PubMed/Medline | 16.02.18 | 209 |  |  |  |  |
| Embase | 16.02.18 | 248 |  |  |  |  |
| PsycInfo | 16.02.18 | 131 |  |  |  |  |
| Epistemonikos | 16.02.18 | 90 |  |  |  |  |
| Global Health Library | 16.02.18 | 33 |  |  |  |  |

*For people with SMD who are smokers, are non-pharmacological interventions effective to support tobacco cessation?*

| **Database searched** | **Date searched** | **No. of papers** | **Combined number, excluding duplicates** | **No. of relevant Cochrane reviews** | **No. of relevant systematic reviews** | **No. of relevant guidelines** |
| --- | --- | --- | --- | --- | --- | --- |
| Cochrane (& DARE) | 16.02.18 | 168 | 707 | 4  (+ 1 protocol) | 8 | 3 |
| PubMed/Medline | 16.02.16 | 176 |  |  |  |  |
| Embase | 16.02.18 | 253 |  |  |  |  |
| PsycInfo | 16.02.18 | 131 |  |  |  |  |
| Epistemonikos | 16.02.18 | 86 |  |  |  |  |
| Global Health Library | 16.02.18 | 35 |  |  |  |  |

*For people with SMD who are smokers, are pharmacological (including nicotine replacement therapy, bupropion, varenicline) interventions effective to support tobacco cessation?*

*For people with SMD who are smokers, are non-pharmacological interventions effective to support tobacco cessation?*

| **Combined number** | **Combined number, excluding duplicates** | **Combined actual number, excluding duplicates**  **(hand searching)** |
| --- | --- | --- |
| 1434 | 774 | 692 |

**Substance use disorders; drugs and/or alcohol**

*For people with SMD and substance (drug and/or alcohol) use disorder, are pharmacological interventions for substance use disorder effective to support reduction in substance use-related outcomes?*

| **Database searched** | **Date searched** | **No. of papers** | **Combined number, excluding duplicates** | **No. of relevant Cochrane reviews** | **No. of relevant systematic reviews** | **No. of relevant guidelines** |
| --- | --- | --- | --- | --- | --- | --- |
| Cochrane (& DARE) | 19.06.18 | 151 | 2256 |  |  |  |
| PubMed/Medline | 19.06.18 | 689 |  |  |  |  |
| Embase | 19.06.18 | 1368 |  |  |  |  |
| PsycInfo | 19.06.18 | 179 |  |  |  |  |
| Epistemonikos | 19.06.18 | 138 |  |  |  |  |
| Global Health Library | 19.06.18 | 0 |  |  |  |  |

*For people with SMD and substance (drug and/or alcohol) use disorder, are non-pharmacological interventions for substance use disorder effective to support reduction in substance use-related outcomes?*

| **Database searched** | **Date searched** | **No. of papers** | **Combined number, excluding duplicates** | **No. of relevant Cochrane reviews** | **No. of relevant systematic reviews** | **No. of relevant guidelines** |
| --- | --- | --- | --- | --- | --- | --- |
| Cochrane (& DARE) | 19.06.18 | 146 | 2012 |  |  |  |
| PubMed/Medline | 19.06.18 | 493 |  |  |  |  |
| Embase | 19.06.18 | 1337 |  |  |  |  |
| PsycInfo | 19.06.18 | 117 |  |  |  |  |
| Epistemonikos | 19.06.18 | 170 |  |  |  |  |
| Global Health Library | 19.06.18 | 0 |  |  |  |  |

*For people with SMD and substance (drug and/or alcohol) use disorder, are pharmacological interventions for substance use disorder effective to support reduction in substance use-related outcomes?*

*For people with SMD and substance (drug and/or alcohol) use disorder, are non-pharmacological interventions for substance use disorder effective to support reduction in substance use-related outcomes?*

| **Combined number** | **Combined number, excluding duplicates** | **Combined number with search 1** | **Combined actual number (combined with search 1), excluding duplicates**  **(hand searching)** |
| --- | --- | --- | --- |
| 4268 | 2551 | 2610 | 2582 |
