# Supplementary material for: Gaps and challenges: WHO treatment recommendations for tobacco cessation and management of substance use disorders in people with severe mental illness
Source: BMC Psychiatry. 2020 May 14;20:237. doi: 10.1186/s12888-020-02623-y (PMC7227317; doi:10.1186/s12888-020-02623-y)
Supplement: Supplementary file 4 — Additional file 4. PRISMA 2009 Checklist [file 12888_2020_2623_MOESM4_ESM.doc]

| **Section/topic** | **#** | **Checklist item** | **Reported on page #** |
| --- | --- | --- | --- |
| **TITLE** | | |  |
| Title | 1 | Identify the report as a systematic review, meta-analysis, or both. | Not included as the paper is not just a systematic review |
| **ABSTRACT** | | |  |
| Structured summary | 2 | Provide a structured summary including, as applicable: background; objectives; data sources; study eligibility criteria, participants, and interventions; study appraisal and synthesis methods; results; limitations; conclusions and implications of key findings; systematic review registration number. | As above- the process for guideline development are detailed in the abstract |
| **INTRODUCTION** | | |  |
| Rationale | 3 | Describe the rationale for the review in the context of what is already known. | 3-4 |
| Objectives | 4 | Provide an explicit statement of questions being addressed with reference to participants, interventions, comparisons, outcomes, and study design (PICOS). | Tables 2a, 2b |
| **METHODS** | | |  |
| Protocol and registration | 5 | Indicate if a review protocol exists, if and where it can be accessed (e.g., Web address), and, if available, provide registration information including registration number. | See <https://apps.who.int/iris/bitstream/handle/10665/275718/9789241550383-eng.pdf?ua=1> Annex material which details methods undertaken |
| Eligibility criteria | 6 | Specify study characteristics (e.g., PICOS, length of follow-up) and report characteristics (e.g., years considered, language, publication status) used as criteria for eligibility, giving rationale. | See link for full details: <https://apps.who.int/iris/bitstream/handle/10665/275718/9789241550383-eng.pdf?ua=1>  Page 5, 6 |
| Information sources | 7 | Describe all information sources (e.g., databases with dates of coverage, contact with study authors to identify additional studies) in the search and date last searched. | See supplementary material Table 3  Page 5 |
| Search | 8 | Present full electronic search strategy for at least one database, including any limits used, such that it could be repeated. | Page 5, also see supplementary material tables 1-3 |
| Study selection | 9 | State the process for selecting studies (i.e., screening, eligibility, included in systematic review, and, if applicable, included in the meta-analysis). | Page 5-6. Figures 1-3. Supplementary material table 3 |
| Data collection process | 10 | Describe method of data extraction from reports (e.g., piloted forms, independently, in duplicate) and any processes for obtaining and confirming data from investigators. | Page 6 |
| Data items | 11 | List and define all variables for which data were sought (e.g., PICOS, funding sources) and any assumptions and simplifications made. | Data items, including details of funding source are listed at in the GRADE tables and footnotes. See <https://www.who.int/mental_health/evidence/evidence_profiles_severe_mental_disorders.pdf?ua=1>  Page 1-32, 37-39 (Tobacco cessation) [starts on page 2 of uploaded document in link]  Page 1-46, 56-64 (Substance use disorders) [starts on page 146 of uploaded document in link] |
| Risk of bias in individual studies | 12 | Describe methods used for assessing risk of bias of individual studies (including specification of whether this was done at the study or outcome level), and how this information is to be used in any data synthesis. | Risk of bias assessed as part of GRADE assessment for certainty of evidence  Page 6 for methods and how this information was used to inform GDG discussions. Also see:  <https://www.who.int/mental_health/evidence/evidence_profiles_severe_mental_disorders.pdf?ua=1>  Page 1-32, 37-39 (Tobacco cessation) [starts on page 2 of uploaded document in link]  Page 1-46, 56-64 (Substance use disorders) [starts on page 146 of uploaded document in link] |
| Summary measures | 13 | State the principal summary measures (e.g., risk ratio, difference in means). | See page 6. Summary measures listed in GRADE tables. See <https://www.who.int/mental_health/evidence/evidence_profiles_severe_mental_disorders.pdf?ua=1>  Page 1-32, 37-39 (Tobacco cessation) [starts on page 2 of uploaded document in link]  Page 1-46, 56-64 (Substance use disorders) [starts on page 146 of uploaded document in link] |
| Synthesis of results | 14 | Describe the methods of handling data and combining results of studies, if done, including measures of consistency (e.g., I^2^) for each meta-analysis. | Methods for handling data and synthesis page 5-6  No meta analysis performed |

Page 1 of 2

| **Section/topic** | **#** | **Checklist item** | **Reported on page #** |
| --- | --- | --- | --- |
| Risk of bias across studies | 15 | Specify any assessment of risk of bias that may affect the cumulative evidence (e.g., publication bias, selective reporting within studies). | pg 5-6 |
| Additional analyses | 16 | Describe methods of additional analyses (e.g., sensitivity or subgroup analyses, meta-regression), if done, indicating which were pre-specified. | Additional analyses not done |
| **RESULTS** | | |  |
| Study selection | 17 | Give numbers of studies screened, assessed for eligibility, and included in the review, with reasons for exclusions at each stage, ideally with a flow diagram. | Figures 2-3 |
| Study characteristics | 18 | For each study, present characteristics for which data were extracted (e.g., study size, PICOS, follow-up period) and provide the citations. | See <https://www.who.int/mental_health/evidence/evidence_profiles_severe_mental_disorders.pdf?ua=1>  Page 1-32, 37-39 (Tobacco cessation) [starts on page 2 of uploaded document in link]  Page 1-46, 56-64 (Substance use disorders) [starts on page 146 of uploaded document in link] |
| Risk of bias within studies | 19 | Present data on risk of bias of each study and, if available, any outcome level assessment (see item 12). | See <https://www.who.int/mental_health/evidence/evidence_profiles_severe_mental_disorders.pdf?ua=1>  Page 1-32, 37-39 (Tobacco cessation) [starts on page 2 of uploaded document in link]  Page 1-46, 56-64 (Substance use disorders) [starts on page 146 of uploaded document in link] |
| Results of individual studies | 20 | For all outcomes considered (benefits or harms), present, for each study: (a) simple summary data for each intervention group (b) effect estimates and confidence intervals, ideally with a forest plot. | See <https://www.who.int/mental_health/evidence/evidence_profiles_severe_mental_disorders.pdf?ua=1>  Page 1-32, 37-39 (Tobacco cessation) [starts on page 2 of uploaded document in link]  Page 1-46, 56-64 (Substance use disorders) [starts on page 146 of uploaded document in link]  Forest plot not relevant |
| Synthesis of results | 21 | Present results of each meta-analysis done, including confidence intervals and measures of consistency. | Meta analysis not performed |
| Risk of bias across studies | 22 | Present results of any assessment of risk of bias across studies (see Item 15). | Performed as part of GRADE approach; See: <https://www.who.int/mental_health/evidence/evidence_profiles_severe_mental_disorders.pdf?ua=1>  Page 1-32, 37-39 (Tobacco cessation) [starts on page 2 of uploaded document in link]  Page 1-46, 56-64 (Substance use disorders) [starts on page 146 of uploaded document in link] |
| Additional analysis | 23 | Give results of additional analyses, if done (e.g., sensitivity or subgroup analyses, meta-regression [see Item 16]). | Additional analyses not performed |
| **DISCUSSION** | | |  |
| Summary of evidence | 24 | Summarize the main findings including the strength of evidence for each main outcome; consider their relevance to key groups (e.g., healthcare providers, users, and policy makers). | See table 3 for WHO recommendations and strength of evidence |
| Limitations | 25 | Discuss limitations at study and outcome level (e.g., risk of bias), and at review-level (e.g., incomplete retrieval of identified research, reporting bias). | Pages 8-9, 10 also  <https://www.who.int/mental_health/evidence/evidence_profiles_severe_mental_disorders.pdf?ua=1>  Page 1-32, 37-39 (Tobacco cessation) [starts on page 2 of uploaded document in link]  Page 1-46, 56-64 (Substance use disorders) [starts on page 146 of uploaded document in link] |
| Conclusions | 26 | Provide a general interpretation of the results in the context of other evidence, and implications for future research. | Page 10-11 |
| **FUNDING** | | |  |
| Funding | 27 | Describe sources of funding for the systematic review and other support (e.g., supply of data); role of funders for the systematic review. | Page 13 |

*From:*  Moher D, Liberati A, Tetzlaff J, Altman DG, The PRISMA Group (2009). Preferred Reporting Items for Systematic Reviews and Meta-Analyses: The PRISMA Statement. PLoS Med 6(7): e1000097. doi:10.1371/journal.pmed1000097

For more information, visit: **www.prisma-statement.org**.

Page 2 of 2
